# Targeting NADPH oxidase 2 suppresses the growth of esophageal squamous cell carcinoma by regulating BTG2 pathway

**DOI:** 10.1007/s12672-025-04345-7

**Published:** 2025-12-24

**Authors:** Xiao-Jie Liu, Can-Lin Yang, Yun-Lei Zhang, Jun-Xing Huang

**Affiliations:** 1 Department of Oncology, The Affiliated Taizhou People’s Hospital of Nanjing Medical University, Taizhou School of Clinical Medicine, Nanjing Medical University, Taizhou, Jiangsu China; 2 Graduate School, Dalian Medical University, Dalian, Liaoning China; 3 Department of Oncology, Yixing Hospital Affiliated to Medical College of JiangSu University, Yixing, Jiangsu China

**Keywords:** Esophageal squamous cell carcinoma, NADPH oxidase 2, B cell translocation gene 2, Prognosis, Biomarker

## Abstract

**Background:**

Nicotinamide adenine dinucleotide phosphate (NADPH) oxidase 2 (NOX2) plays a carcinogenic role in various tumors. Recent study has reported that the carcinogenic mechanism of NOX2 in esophageal squamous cell carcinoma (ESCC) may be associated with the regulation of B-cell translocation gene 2 (BTG2). This study aims to clarify the role of NOX2 in the progression of ESCC.

**Materials and methods:**

NOX2 was knocked down in ESCC cell lines via siRNA transfection to assess its impact on cell function and BTG2 regulation. Tumor tissues from 66 ESCC patients were analyzed for NOX2 and BTG2 expression using immunohistochemistry to determine their clinical significance.

**Results:**

NOX2 deficiency inhibited ESCC cell proliferation, promoted apoptosis, and caused G1 phase arrest. Following NOX2 knockdown, BTG2 mRNA expression significantly increased in TE1 and KYSE30 cells, and a notable rise in BTG2 protein expression was observed in KYSE30 cells. Immunohistochemical analysis revealed that NOX2 expression was significantly higher in tumor tissues compared to adjacent non-cancerous tissues, while BTG2 expression was markedly lower in tumor tissues. High NOX2 expression correlated with poor prognosis, whereas high BTG2 expression indicated better outcomes. Multivariate analysis indicated that abnormal NOX2 and BTG2 expressions are independent prognostic factors for ESCC.

**Conclusion:**

The carcinogenic role of NOX2 in ESCC may be associated with the regulation of BTG2 expression. The aberrant expressions of NOX2 and BTG2 are associated with the prognosis of patients with ESCC, suggesting that NOX2 and BTG2 could serve as potential biomarkers and therapeutic targets.

**Supplementary Information:**

The online version contains supplementary material available at 10.1007/s12672-025-04345-7.

## Introduction

Cancer remains a leading cause of global mortality, characterized by uncontrolled cell proliferation, evasion of growth suppressors, and metabolic reprogramming. Current therapeutic strategies encompass surgery, radiotherapy, chemotherapy, and more recently, targeted therapy and immunotherapy, which have revolutionized the management of advanced malignancies [1]. Esophageal cancer (EC) is a common malignant tumor of the digestive tract, ranking 8th in incidence and 6th in mortality worldwide [2]. Asia has the highest incidence of EC, with China accounting for nearly half of the global cases [3]. The pathological types of EC mainly include esophageal squamous cell carcinoma (ESCC) and adenocarcinoma, with ESCC accounting for approximately 90% of EC cases [4]. In recent years, programmed cell death 1 (PD-1) inhibitors have significantly improved the treatment of advanced ESCC and greatly extended patient survival [5]. However, not all ESCC patients benefit from immunotherapy [6, 7]. Therefore, it is essential to explore new therapeutic targets to better understand the tumorigenesis of esophageal cancer and develop new targeted treatment strategies.

Reduced nicotinamide adenine dinucleotide phosphate (NADPH) oxidase (NOX) is a family of transmembrane proteins consisting of seven structurally conserved isoforms, including NOX1-NOX5 and Duox1-2 [8]. NOX family members are distributed across various tissues and participate in many important physiological processes by catalyzing the reduction of oxygen molecules to produce reactive oxygen species (ROS) [9]. Dysregulation of NOXs can lead to a range of severe pathological conditions, such as atherosclerosis, hypertension, diabetic nephropathy, pulmonary fibrosis, and neurodegenerative diseases [10]. Recent studies have reported that NOXs can be activated in various cancers and exhibit carcinogenic characteristics [11].

NOX2 was the first identified member of the NOX family [12]. In gastric cancer, NOX2 has been found to be highly expressed in tumor tissues [13], and the NOX2-ROS axis can mediate the activation of tumorigenesis-associated pathways, including the Akt-mTOR, Stat3, and NF-κB signaling pathways [14]. While in lung cancer, the NOX2/ROS-related signaling pathway is activated within cancer cells to sustain their invasiveness [15]. Similarly, its aberrant expression has been documented in prostate cancer [16], ovarian cancer [17], colon cancer [18], and ESCC [19]. However, no studies have yet explored the expression and physiological function of NOX2 in the progression of ESCC in the Chinese population, and the carcinogenic mechanism of NOX2 remains unclear.

Research has indicated a strong correlation between NOX2 gene expression and the cell cycle regulatory signaling pathways regulated by the B-cell translocation gene 2 (BTG2) family [19]. As an important tumor suppressor gene, BTG2 plays a role in several core biological processes such as cell cycle regulation, apoptosis induction, and DNA damage response [20]. Directly targeting tumor cells and modulating the tumor microenvironment represent two crucial avenues in cancer therapy [21]. Whether targeting NOX2 and BTG2 can simultaneously influence both cell-autonomous tumor behaviors and broader stromal interactions represents a highly compelling research topic. In this study, we employed immunohistochemistry (IHC) to analyze the expression of NOX2 and BTG2 in ESCC tissues from the Chinese population to determine the association of these two targets with the prognosis of ESCC patients. Additionally, we investigated the impact of NOX2 on the biological functions of ESCC cells and its relationship with the BTG2 pathway to elucidate the connection between NOX2 expression and cancer progression.

## Materials and methods

### Cell lines and cell culture

The ESCC cell lines TE1, TE10, TE13, KYSE30 and KYSE150 and human normal esophageal epithelial cell line HET1A were purchased from Meixuan Company (Shanghai, China). All experiments were performed with mycoplasma-free cells. All the cell lines were cultured in an RPMI 1640 medium (Cat. #10–040-CRV, Corning, NY, USA) supplemented with 10% fetal bovine serum (FBS; Cat. #Sh30084.03, Hyclone, South Logan, UT, USA), 100 U/mL penicillin, and 100 µg/mL streptomycin (Cat. #15140112, Gibco, Grand Island, NY, USA). Cells were incubated at 37℃ in a 5% CO2-95% air atmosphere.

### Quantitative reverse transcription polymerase chain reaction (RT-qPCR)

Total RNA was extracted from cells using an RNAiso plus kit (Cat. #9108, TAKARA, Tokyo, Japan). cDNA was synthesized using HiFiScript cDNA Synthesis Kit (Cat. #Cw2569, ComWin Biotech, Beijing, China). RT-qPCR was performed on an Applied Biosystems 7500 Real-Time PCR instrument (ABI, Carlsbad, CA, USA) using SYBR Premix Ex Taq (Cat. #MX200017, Meixuan, Shanghai, China). The special primers were used as followed: NOX2 (forward), 5’-TGCCAGTCTGTCGAAATCTGC-3’; NOX2 (reverse), 5’-ACTCGGGCATTCACACC-3’; GAPDH (forward), 5’-GCACCGTCAAGGCTGAGAAC-3’; GAPDH (reverse), 5’-TGGTGAAGACGCCAGTA-3’. RT-qPCR conditions were 95℃ for 5 min, 40 cycles of 95℃ for 15 s and 60℃ for 30 s, and 60℃ for 2 min. All RT-qPCR experiments were performed in triplicates. RT-qPCR was performed to detect the mRNA expression levels of the target genes. ∆Ct values were used to determine absolute expression, and ∆∆Ct values were used to determine relative expression as fold changes occurred. Using the 2−∆∆Ct method, the relative expression levels of the target genes for each sample were normalized to those of the endogenous control GAPDH.

### Western blotting

Western blotting was performed as previously described [22]. Briefly, equal amounts of 20 µg proteins extracted from cells were subjected to SDS-PAGE and then electrically transferred to polyvinylidene difluoride (PVDF) membranes. The membrane was blocked with 5% BSA for 1 h at room temperature and then incubated overnight at 4℃ with the primary antibodies. After washing three times with TBST (0.1% Tween in TBS), the membrane was incubated with secondary antibodies for 2 h at room temperature. The band intensity was estimated using Image Pro Plus 6.0 (Media Cybernetic Inc, USA). The following antibodies were used in Western blotting experiments: mouse anti-GAPDH (Cat. #D190090, 1:5000, BBI, Shanghai, China), rabbit anti-NOX2 (Cat. #19013-1-AP, 1:1000, Proteintech, Chicago, IL, USA), rabbit anti-BTG2 (Cat. #22339-1-AP, 1:1000, Proteintech, Chicago, IL, USA), HRP-conjugated Goat Anti-Rabbit IgG (Cat. #D110058, 1:5000, BBI, Shanghai, China), HRP-conjugated Goat Anti-Mouse IgG (Cat. #D110087, 1:5000, BBI, Shanghai, China). GAPDH was used as a loading control for Western blot analysis. Its expression remained stable across all experimental conditions, and it has no known functional interplay with the proteins of interest (NOX2 and BTG2), supporting its suitability as a normalization control.

### Small interfering RNA (siRNA) transfection

Three siRNA sequences were designed for instantaneous transfection of TE1 and KYSE30 cells. Lipofectamine™ 2000 Transfection Reagent (Cat. #11668, Invitrogen, Carlsbad, CA, USA) was performed for all siRNA transfection procedures at a final siRNA concentration of 100 pmol/l according to the manufacturer’s instructions. Cells transfected with si-NC were considered as the negative control group and cells without transfection were considered as the blank control. 48 h post transfection cells were harvested for RT-qPCR. The siRNA with the highest transfection rate was used for subsequent experimental transfection.

### Cell proliferation assay

Cell proliferation was analyzed 48 h and 72 h after siRNA transfection using a CCK-8 kit (Cat. #G4103, Servicebio, Wuhan, China). According to the manufacturer’s instructions, TE1 and KYSE30 were seeded in a 96-well culture plate and incubated for 48 h and 72 h, respectively. After adding 10 µl of CCK-8 reagent to each well, the cells were further incubated for 1 h. Then the absorbance at 450 nm was measured using a microplate reader. Each assay was performed in triplicate.

### Annexin V staining

Cell apoptosis was analyzed using the Annexin V method. After siRNA transfection for 48 h, TE1 and KYSE30 cells were washed with pre-cooled PBS, digested with EDTA-free trypsin, and then stained with an Annexin V-FITC/PI kit (Cat. #G1514, Servicebio, Wuhan, China) according to the manufacturer’s protocol. The cell apoptosis was analyzed using an EXFOLW-206 Flow Cytometry (DAKEWE, Shenzhen, China). Each assay was performed in triplicate.

### Cell cycle analysis

After siRNA transfection for 48 h, TE1 and KYSE30 cells were harvested, washed using PBS three times, and then fixed in pre-cooled 70% ethanol at 4℃ overnight. After incubation with propidium iodide (PI) solution (Cat. #1700, Servicebio, Wuhan, China) for 30 min at 37℃ in the dark, the cells were measured using an EXFOLW-206 Flow Cytometry (DAKEWE, Shenzhen, China). Each assay was performed in triplicate.

### Sampling

This retrospective study enrolled 66 ESCC patients who underwent radical esophagectomy at Taizhou People’s Hospital between January 2019 and December 2020. Inclusion criteria: (1) histopathologically confirmed ESCC; (2) availability of paired tumor and paracancerous tissue specimens (resection margin ≥ 5 cm); (3) complete clinicopathological records. Exclusion criteria: (1) synchronous malignancies; (2) prior anticancer therapies (including chemoradiotherapy, target therapy, or immune checkpoint inhibitors). Clinicopathological parameters were evaluated according to the American Joint Committee on Cancer (AJCC) 8th edition tumor-node-metastasis (TNM) staging system. Baseline data were extracted from the hospital information system (HIS). Postoperative follow-up was conducted through tri-monthly telephone interviews until August 2022, with a median follow-up duration of 38 months. Overall survival (OS) was defined as the interval from curative resection to all-cause mortality.

### IHC

Formalin-fixed paraffin-embedded tissue blocks were sectioned into 3–4 μm slices. After standard deparaffinization through graded ethanol series and rehydration in deionized water, the slides were immersed in sodium citrate buffer (10 mM, pH 6.0) and subjected to microwave-mediated heating at 95–100 °C for 15 min. Sections were blocked with 5% BSA for 30 min and subsequently incubated overnight at 4 °C with diluted primary antibodies (rabbit anti-NOX2, Cat. #19013-1-AP, 1:100, Proteintech, Chicago, IL, USA; rabbit anti-BTG2, Cat. #22339-1-AP, 1:100, Proteintech, Chicago, IL, USA). Secondary antibody (HRP-conjugated Goat Anti-rabbit IgG, Cat. #D110058, 1:100, BBI, Shanghai, China) was applied under dark conditions at room temperature for 30 min, followed by 3,5-diaminobenzidine chromogenic development and hematoxylin counterstaining. Positive staining was defined by cytoplasmic yellowish-brown granules. Three high magnification fields (200×) of view were randomly selected for image acquisition in each section. Optical density (OD) and positive area percentage were quantified using Image Pro Plus 6.0 software (Media Cybernetic Inc, USA). The expression levels of NOX2 and BTG2 were determined through calculation of the average positive index (OD × positive area percentage). Threshold stratification for high versus low expression was established using median positive index values derived from comparative analysis of tumor and paracancerous tissues [23].

### Statistical analysis

All statistical analyses were performed using GraphPad Prism software version 6.01 (La Jolla, CA, USA). Analysis of variance and chi-square test were used for statistical analysis. For survival analysis, survival curves were plotted using the Kaplan–Meier method and evaluated for statistical significance using a log-rank test. Cox regression analysis was used to assess prognostic factors. Univariate *P* < 0.05 was further included in multivariate model for analysis. A *P*-value of less than 0.05 was considered statistically significant.

## Results

### NOX2 expression in human ESCC cell lines

**Fig. 1:**
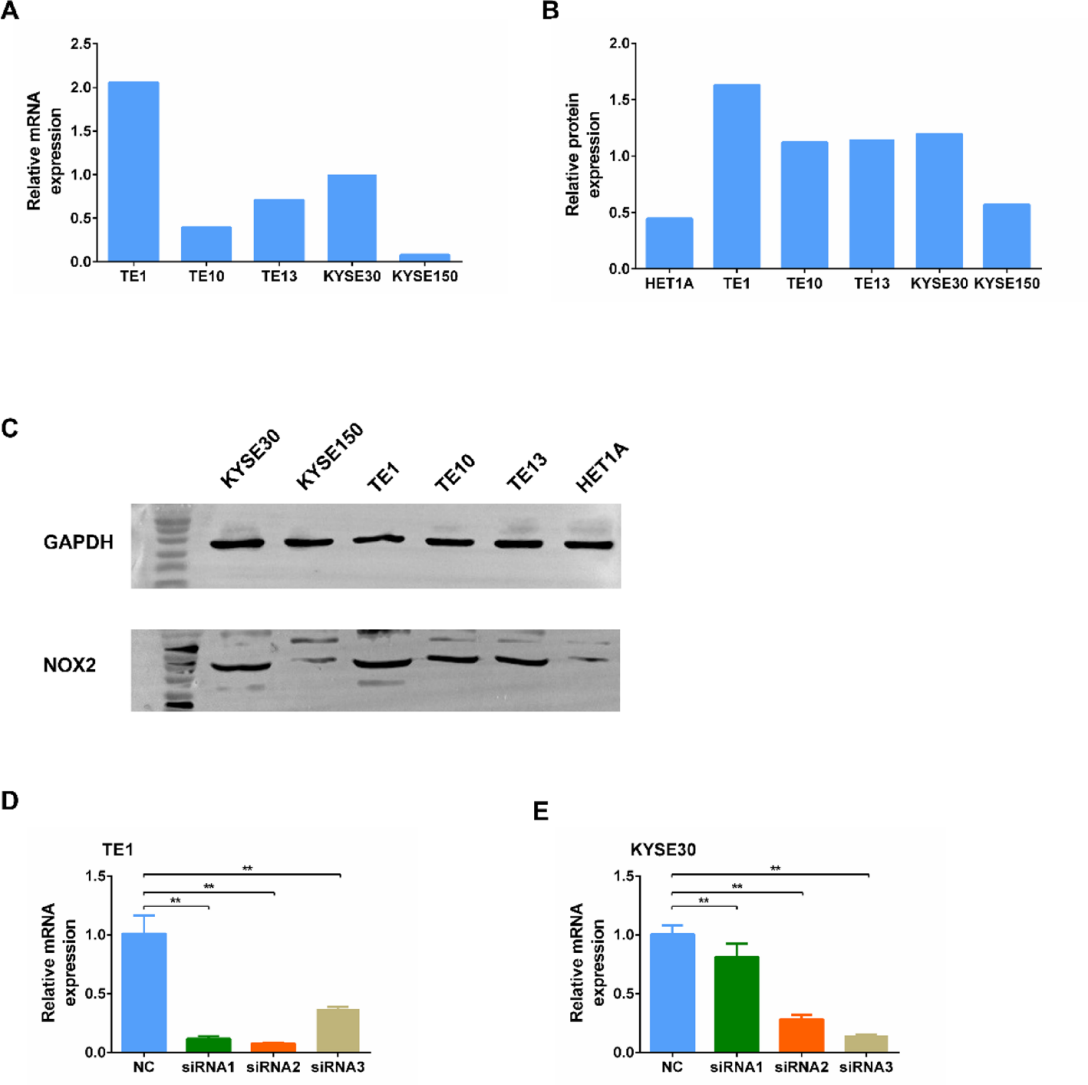
Expression of NOX2 in ESCC cells and transfection of siNOX2 in ESCC cells. **A** mRNA expression levels of NOX2 in five human ESCC cell lines (*n* = 3). **B** Statistical analysis of the Western blot results presented in (C). **C** Western blot analysis detecting the protein expression levels of NOX2 in five human ESCC cell lines and human normal esophageal epithelial cell line HET1A (*n* = 1). **D** mRNA expression levels of NOX2 after transfection of three different siRNAs in TE1 cells (*n* = 3). **E** mRNA expression levels of NOX2 after transfection of three different siRNAs in KYSE30 cells (*n* = 3). **, *P* < 0.01

We selected five human ESCC cell lines: TE1, TE10, TE13, KYSE30, and KYSE150, and assessed the mRNA and protein expression levels of NOX2 in these cell lines using qRT-PCR and Western blotting. Among them, TE1 and KYSE30 cells exhibited the highest levels of NOX2 mRNA and protein expression (Fig. 1A–C). Therefore, TE1 and KYSE30 cells were selected for subsequent experiments to investigate the impact of NOX2 knockdown on ESCC tumor progression. The transfection efficiency of three siRNAs was evaluated using qRT-PCR, revealing that siRNA2 had the highest transfection efficiency in TE1 cells, while siRNA3 showed the highest transfection efficiency in KYSE30 cells (Fig. 1D and E).

### NOX2 depletion suppresses ESCC cell proliferation

**Fig. 2:**
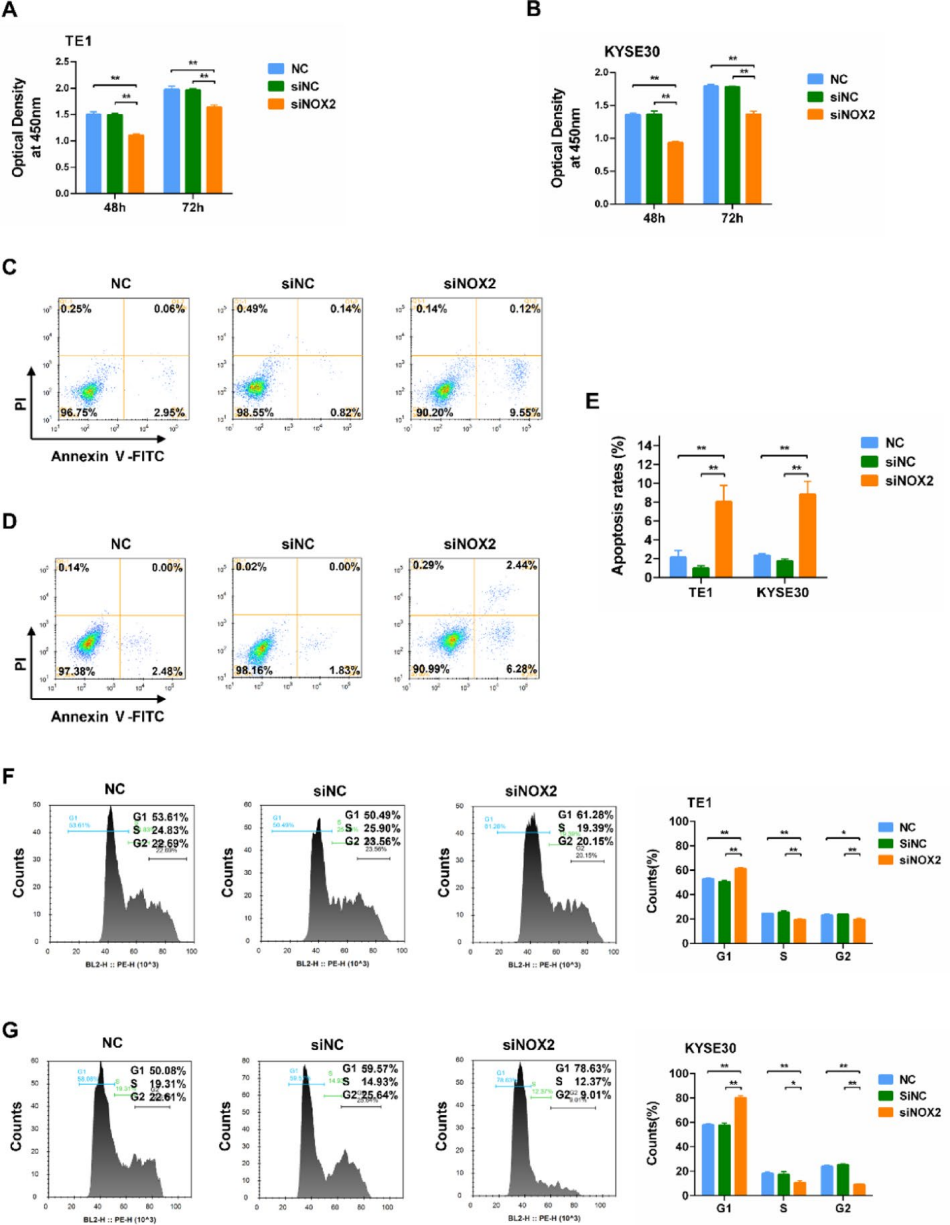
Effects of NOX2 knockdown on the proliferation, apoptosis, and cell cycle in ESCC Cells. **A**,** B** Proliferation changes in TE1 (**A**) and KYSE30 (**B**) cells at 48 h and 72 h after NOX2 knockdown (*n* = 3). **C**,** D** Annexin V/PI assay detecting apoptosis in TE1 (**C**) and KYSE30 (**D**) cells at 48 h after NOX2 knockdown (*n* = 3). **E** Statistical analysis of the Annexin V/PI assay results presented in **C**,** D**. **F**,** G** Analysis for the change of cell cycle in TE1 (**F**) and KYSE30 (**G**) cells at 48 h after NOX2 knockdown (*n* = 3). *, *P* < 0.05; **, *P* < 0.01

The results of the CCK-8 assay indicated that NOX2 siRNA knockdown significantly inhibited the proliferation of TE1 and KYSE30 cells at 48 h and 72 h post-transfection (Fig. 2A and B).

### NOX2 depletion promotes ESCC cell apoptosis and cycle arrest

The results of the Annexin V/PI assay demonstrated that NOX2 siRNA knockdown significantly promoted the apoptosis of TE1 and KYSE30 cells at 48 h post-transfection (Fig. 2C–E). Cell cycle analysis revealed that NOX2 knockdown resulted in G1 phase cell cycle arrest in both TE1 and KYSE30 cells (Fig. 2F and G). These results implied that NOX2 functioned in regulating the apoptosis and cell cycle of ESCC cells.

### NOX2 depletion promotes the expression of BTG2

**Fig. 3:**
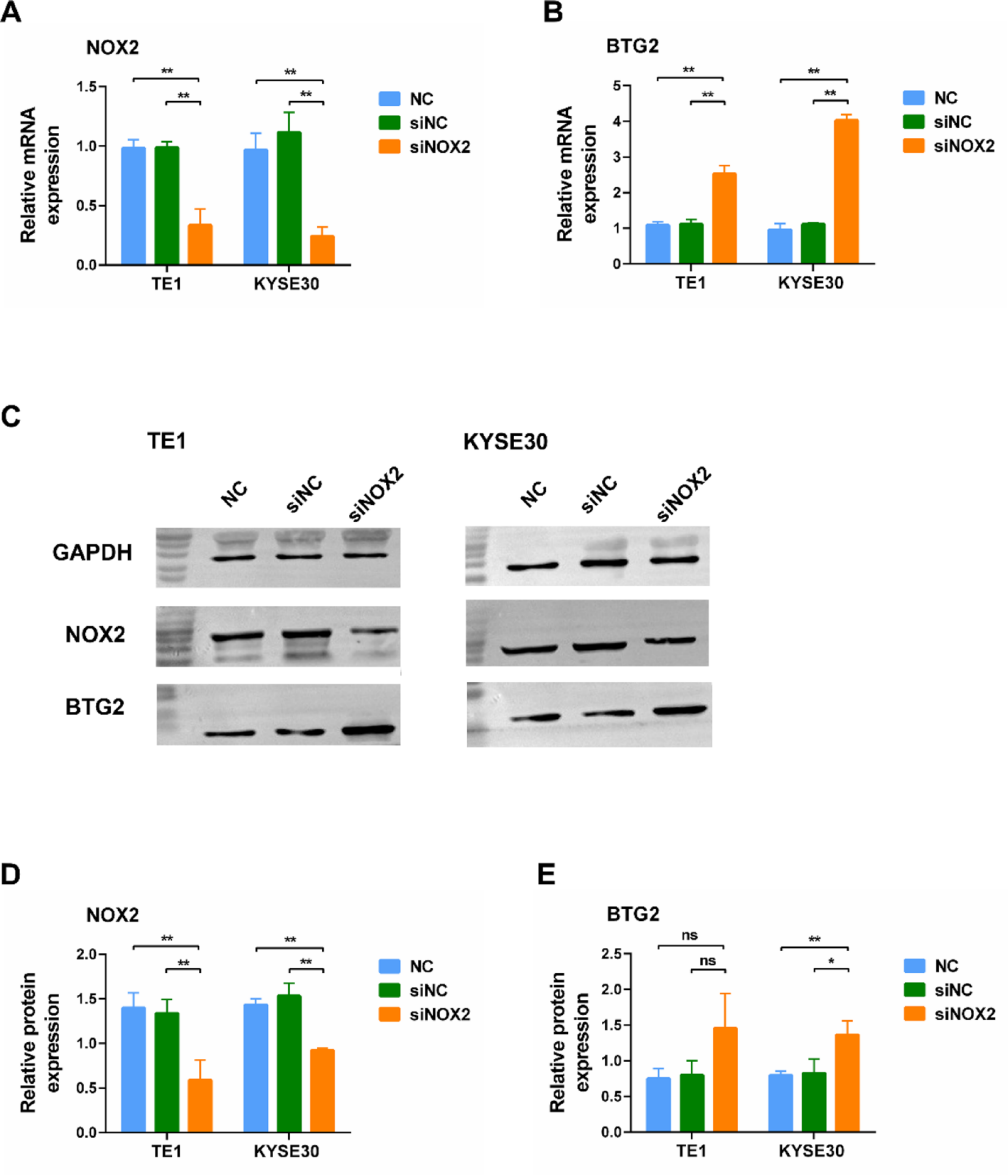
Expression of BTG2 in ESCC cells after 48 h of siNOX2 transfection. **A**,** B** mRNA expression of NOX2 (**A**) and BTG2 (**B**) in TE1 and KYSE30 cells following NOX2 knockdown (*n* = 3). **C** Western blot analysis detecting the protein expression of NOX2 and BTG2 in TE1 and KYSE30 cells after NOX2 knockdown (*n* = 3). **D**,** E** Statistical analysis of the Western blot results presented in **C**. ns, no significance. *, *P* < 0.05; **, *P* < 0.01

We utilized qRT-PCR and Western blot assays to examine the effects of NOX2 knockdown on BTG2 expression in ESCC cells 48 h post-transfection. The qRT-PCR results indicated that BTG2 mRNA levels were significantly elevated in TE1 and KYSE30 cells following NOX2 knockdown compared to the control group (Fig. 3B). Western blot analysis showed that NOX2 protein levels significantly decreased in both TE1 and KYSE30 cells post-NOX2 knockdown, while BTG2 protein levels were significantly increased in KYSE30 cells compared to the control group (Fig. 3C–E). Although BTG2 protein levels in TE1 cells showed an upward trend, the difference was not statistically significant (*P* > 0.05).

### IHC analysis of NOX2 and BTG2 in ESCC tissues

**Fig. 4:**
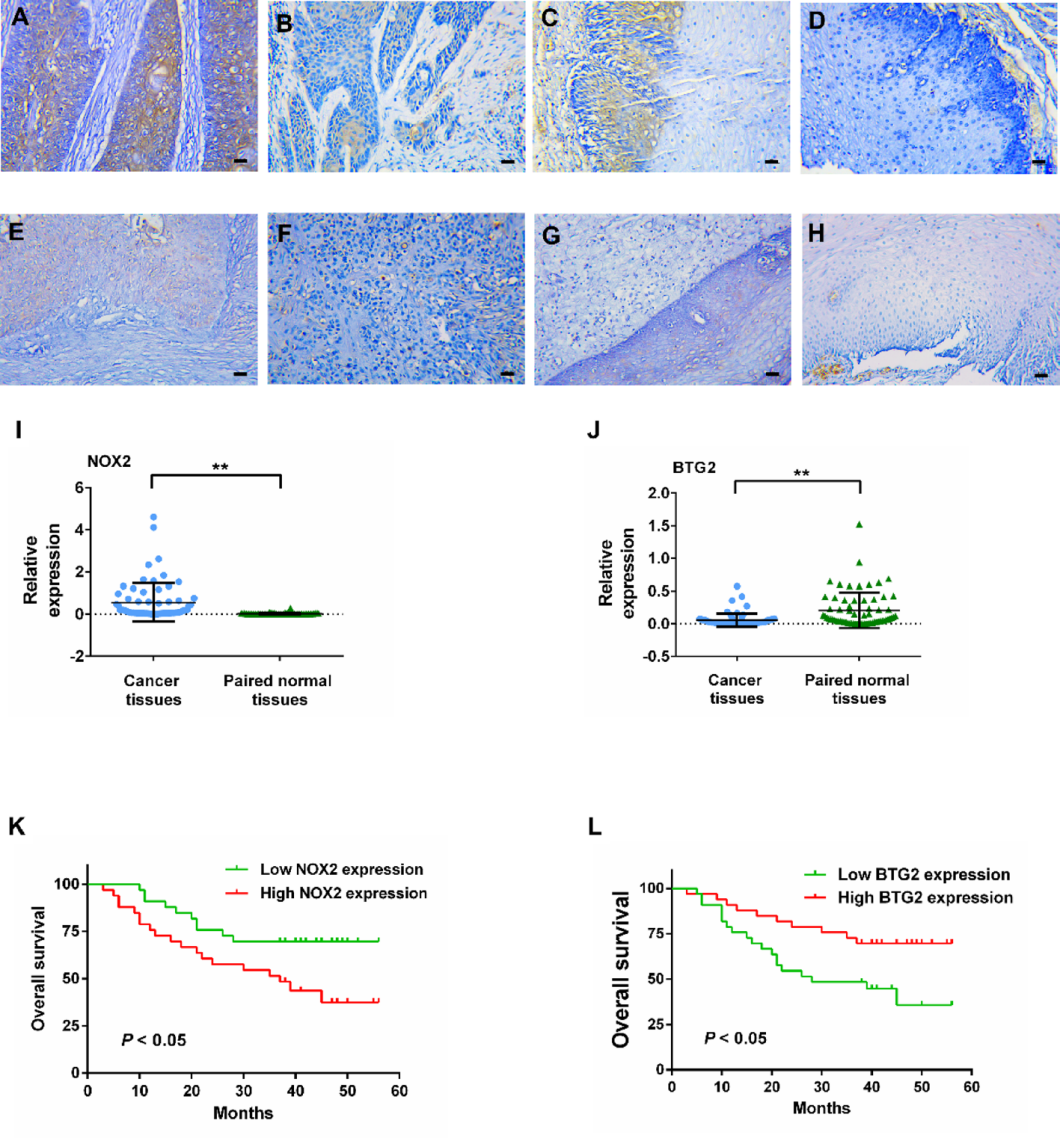
IHC analysis and survival curves for the protein expression of NOX2 and BTG2 in ESCC samples. **A** High expression of NOX2 in ESCC tumor tissues. **B** Low expression of NOX2 in ESCC tumor tissues. **C** High expression of NOX2 in ESCC adjacent tissues. **D** Low expression of NOX2 in ESCC adjacent tissues. **E** High expression of BTG2 in ESCC tumor tissues. **F** Low expression of BTG2 in ESCC tumor tissues. **G** High expression of BTG2 in ESCC adjacent tissues. **H** Low expression of BTG2 in ESCC adjacent tissues. **I** NOX2 expression in ESCC tissues and adjacent tissues (Wilcoxon signed-rank test; Z = −6.69; **, *P* < 0.01). **J** BTG2 expression in ESCC tissues and adjacent tissues (Wilcoxon signed-rank test; Z = 5.07; **, *P* < 0.01). **K**,** L** Kaplan − Meier survival curves for overall survival of ESCC patients with different NOX2 expression (**K**) and BTG2 expression (**L**). Magnification, ×200. Scale bars, 100 μm

IHC staining of NOX2 and BTG2 in ESCC tissue samples revealed that cells with high expression of NOX2 and BTG2 appeared brown under the microscope, while cells with low expression appeared light yellow or colorless. Both NOX2 and BTG2 were localized to the cell membrane and/or cytoplasm, with expression observed in both ESCC tumor cells and adjacent non-cancerous cells (Fig. 4A–H). The expression of NOX2 in tumor tissues was significantly higher than that in adjacent tissues (Fig. 4I), whereas BTG2 expression in tumor tissues was significantly lower than that in adjacent tissues (Fig. 4J).

We evaluated the expression levels of NOX2 and BTG in primary ESCC specimens from 66 patients from eastern China. Among these patients, 47 (71.2%) were men and 19 (28.8%) were women, with a mean age of 67.6 years (range 44–80). According to the 8th AJCC staging, 34 (51.5%) patients were classified as stage I–II and 32 (48.5%) as stage III–IV. Other clinicopathological characteristics are provided in Table 1. The mean follow-up time was 38 months (range 3–56 months). Specific clinicopathological data are presented in Supplementary data 1.

**Table 1:** Correlation of NOX2 and BTG2 expression with clinicopathological characteristics of ESCC patients

Features	Cases (*n*)	Expression of NOX2		χ^2^	*P* value	Expression of BTG2		χ^2^	*P* value
		Low	High			Low	High		
Gender									
Male	47	21 (44.7%)	26 (55.3%)	1.848	0.174	21 (44.7%)	26 (55.3%)	1.848	0.174
Female	19	12 (63.2%)	7 (36.8%)			12 (63.2%)	7 (36.8%)		
Age (years)									
< 60	12	5 (41.7%)	7 (58.3%)	0.407	0.523	4 (33.3%)	8 (66.7%)	1.630	0.202
≥ 60	54	28 (51.9%)	26 (48.1%)			29 (53.7%)	25 (46.3%)		
Length of tumor (cm)									
≤ 4	44	22 (50.0%)	22 (50.0%)	0	1	22 (50.0%)	22 (50.0%)	0	1
> 4	22	11 (50.0%)	11 (50.0%)			11 (50.0%)	11 (50.0%)		
Tumor location									
Upper Middle	45	20 (44.4%)	25(55.6%)	1.746	0.186	25 (55.6%)	20 (44.4%)	1.746	0.186
Lower paragraph	21	13 (61.9%)	8 (38.1%)			8 (38.1%)	13 (61.9%)		
Differentiation									
Poorly	28	16 (57.1%)	12 (42.9%)	0.992	0.319	19 (67.9%)	9 (32.1%)	6.203	0.013*
Well + moderately	38	17 (44.7%)	21 (55.3%)			14 (36.8%)	24 (63.2%)		
Vascular infiltration									
None	26	12 (46.2%)	14 (53.8%)	0.254	0.614	13 (50.0%)	13 (50.0%)	0	1
Yes	40	21 (52.5%)	19 (47.5%)			20 (50.0%)	20 (50.0%)		
Nerve invasion									
None	23	9 (39.1%)	14 (60.9%)	1.668	0.196	11 (47.8%)	12 (52.2%)	0.067	0.796
Yes	43	24 (55.8%)	19 (44.2%)			22 (51.2%)	21 (48.8%)		
T Stage									
1	7	2 (28.6%)	5 (71.4%)	0.639	0.424	2 (28.6%)	5 (71.4%)	0.639	0.424
2–3	59	31 (52.5%)	28 (47.5%)			31 (52.5%)	28 (47.5%)		
Lymph node metastasis									
None	37	16 (43.2%)	21 (56.8%)	1.538	0.215	18 (48.6%)	19 (51.4%)	0.062	0.804
Yes	29	17 (58.6%)	12 (41.4%)			15 (51.7%)	14 (48.3%)		
TNM Staging									
Phase I - II	34	16 (47.1%)	18 (52.9%)	0.243	0.622	15 (44.1%)	19 (55.9%)	0.971	0.325
Phase III	32	17 (53.1%)	15 (46.9%)			18 (56.3%)	14 (43.7%)		
BTG2 expression									
Low	33	19 (57.6%)	14 (42.4%)	1.515	0.218	–	–	–	–
High	33	14 (42.4%)	19 (57.6%)			–	–		

*P < 0.05

Using the median of the average positive index of NOX2 and BTG2 as two cut-off points, we divided the 66 patients into a NOX2 high expression group (*n* = 33), NOX2 low expression group (*n* = 33), BTG2 high expression group (*n* = 33), and BTG2 low expression group (*n* = 33). As shown in Table 1, the clinicopathological factors of the patients, including gender, age, tumor length, tumor location, degree of tumor differentiation, presence or absence of vascular infiltration, nerve invasion, and lymph node metastasis (LNM), T-stage, TNM stage, and expression levels of NOX2 and BTG2, were subjected to chi-square tests.

Statistical analysis revealed that the expression of NOX2 in tumor tissues was not significantly associated with any clinicopathological factors. However, the expression level of BTG2 was significantly correlated with the degree of tumor differentiation (*P* = 0.013), suggesting that patients with low BTG2 expression were more likely to exhibit poorly differentiated tumors. Furthermore, no significant correlation was observed between NOX2 and BTG2 expression levels.

### Expression of NOX2 and BTG2 is associated with the prognosis of ESCC patients

The association of NOX2 and BTG2 expression with the OS of ESCC patients was analyzed using Kaplan-Meier survival curves and log-rank tests. As of the follow-up cutoff date, 29 patients had died, while 37 were still alive. The results indicated that patients with high NOX2 expression had a shorter OS compared to those with low expression (median OS: high NOX2 expression group 37 months VS low NOX2 expression group not reached, HR = 2.34, 95%CI: 1.12–4.9, *P* = 0.024), with a 3-year survival rate of 51.51% (compared to 69.7% in the low expression group) (Fig. 4K). Conversely, patients with high BTG2 expression had a longer OS than those with low expression (median OS: high BTG2 expression group not reached VS low BTG2 expression group 28, HR = 0.42, 95%CI: 0.20–0.86, *P* = 0.02), with a 3-year survival rate of 72.7% (compared to 48.5% in the low expression group) (Fig. 4L).

### Cox proportional hazard regression model

Factors affecting the prognosis of the 66 patients with ESCC were selected for the multivariate Cox proportional hazard regression model. All significant factors (*P* < 0.05) from the univariate analysis were included in the multivariate model. Univariate analysis revealed that gender, tumor size, vascular invasion, nerve invasion, T-stage, N-stage, and TNM stage were not associated with OS in ESCC patients. However, age, lesion location, degree of differentiation, NOX2 and BTG2 expression levels were significantly associated with OS in ESCC patients and were selected for further multivariate analysis. The results of the multivariate analysis indicated that abnormal expression NOX2 and BTG2 were independent prognostic factors for ESCC patients (Table 2).

**Table 2:** Univariate and multivariate analyses of clinicopathological features for the prognosis in ESCC patients

Features	Univariate analysis		*P* value	Multivariate analysis		*P* value
	HR	95%CI		HR	95%CI	
Gender (Male VS Female)	1.108	0.490–2.506	0.805			
Age (≥ 60 years VS < 60 years)	8.073	1.096–59.486	0.040*	5.512	0.718–42.342	0.101
Length of tumor (> 4 cm VS ≤ 4 cm)	1.097	0.510–2.359	0.813			
Tumor location (Lower VS Upper Middle)	0.360	0.137–0.946	0.038*	0.500	0.186–1.341	0.168
Differentiation (Well + moderately VS Poorly)	0.425	0.202–0.894	0.024*	0.488	0.222–1.072	0.074
Lymph node metastasis (Yes VS None)	1.736	0.828–3.638	0.144			
Vascular infiltration (Yes VS None)	1.599	0.726–3.522	0.244			
Nerve invasion (Yes VS None)	1.340	0.610–2.945	0.466			
T Stage (T2–3 VS T1)	1.850	0.439–7.789	0.402			
TNM Staging (Phase III VS I - II)	1.187	0.571–2.468	0.646			
NOX2 expression (High VS Low)	2.346	1.089–5.054	0.029*	3.517	1.564–7.909	0.002*
BTG2 expression (High VS Low)	0.41	0.192–0.895	0.025*	0.391	0.174–0.882	0.024*

*P < 0.05

## Discussion

In various tumors, the expression of NOX2 is associated with biological behaviors such as cell proliferation and apoptosis. In prostate cancer, NOX2 participates in tumor development by promoting tumor cell proliferation and angiogenesis [16]. Wang et al. reported that Aloin could inhibit the proliferation and migration of gastric cancer cells by downregulating NOX2-ROS-mediated activation of the Akt-mTOR, Stat3, and NF-κB signaling pathways [14]. Takiguchi et al.. demonstrated that NOX2 knockdown significantly inhibited tumor cell proliferation, promoted G1-S phase cell cycle arrest, and reduced the number of migrating and invasive cells in colorectal cancer [18]. In osteosarcoma, NOX2 deficiency was associated with decreased tumor cell viability and increased apoptosis [24]. Shimizu et al. reported that NOX2 knockdown could inhibit cell proliferation, promoting apoptosis and cell cycle arrest in ESCC [19]. Up until the submission of this manuscript, this study is the first to investigate the expression and physiological function of NOX2 in ESCC in the Chinese population. The results of this study are consistent with previous findings: NOX2 deficiency can inhibit ESCC cell proliferation, promote apoptosis, and induce cell cycle arrest, suggesting that NOX2 plays a role in promoting tumor growth in ESCC.

Most studies indicate that the expression level of NOX2 has a limited correlation with clinical pathological factors in tumor patients. Shimizu et al.. indicated that high expression of NOX2 in ESCC tissues was significantly associated only with pathological T staging, while no significant correlations were observed with other factors [19]. A study on colon cancer showed that the expression of NOX2 was not significantly associated with clinical pathological factors [18]. Additionally, findings from a study on osteosarcoma revealed that the overexpression of NOX2 was only related to tumor size and location [25]. Notably, the expression of NOX2 in gastric cancer was associated with the site of occurrence, degree of differentiation, lymph node metastasis, vascular invasion, clinical staging, and TNM staging [13]. In this study, the expression of NOX2 in ESCC tissues showed no significant correlations with clinical pathological factors. This may be due to the insufficient sample size after stratification of the clinical pathological factors, or it could be attributed to greater heterogeneity among different tumors.

In different types of tumors, the correlation between NOX2 expression and prognosis in cancer patients has not yet been clearly established. Studies have reported that in malignant tumors such as gastric cancer [13], colon cancer [18], pancreatic cancer [26], osteosarcoma [25], and ESCC [19], the expression of NOX2 in tumor tissues is higher than that in adjacent non-cancerous tissues. And survival analysis indicates that high expression of NOX2 in tumor tissues is associated with poorer prognosis of cancer patients. Additionally, NOX2 has been identified as an independent prognostic factor in gastric cancer [13], osteosarcoma [25], and ESCC [19]. The findings of this study align closely with previous research: NOX2 is highly expressed in ESCC tumor tissues and serves as an independent prognostic factor. Patients with high NOX2 expression have poorer prognoses, with a 3-year survival rate of only 51.5%. Notably, You et al.. found that the mRNA expression of NOX2 was significantly elevated in gastric cancer tissues, and patients with high NOX2 expression had better prognoses [27]. In tumor tissues of cervical cancer patients infected with human papillomavirus (HPV), NOX2 mRNA levels were significantly elevated, and high NOX2 mRNA levels were notably associated with improved OS [28]. Another study confirmed that NOX2 expression levels were lower in lung adenocarcinoma tumor tissues compared to adjacent normal tissues, with high NOX2 expression correlating with better prognosis in lung adenocarcinoma patients [29]. Therefore, more clinical experimental results are needed to draw more precise conclusions regarding the prognostic impact of NOX2 on malignant tumors.

BTG2 is a member of the BTG/Tob family, which includes six members: BTG1, BTG2/Tis21/PC3, BTG3/ANA, BTG4/PC3B, TOB1/TOB, and TOB2 [30]. BTG2 was the first identified anti-proliferative factor in the BTG/Tob family, playing a key role in cell proliferation, apoptosis, and growth by regulating transcriptional and translational levels [31]. Reduced expression of BTG2 is often associated with malignant cell behavior and poor therapeutic outcome in various tumors [20]. Studies on ESCC have shown that BTG2 expression in tumor tissues is significantly lower than in adjacent normal tissues and is strongly associated with postoperative metastasis, lymph node metastasis, and clinical stage of patients. Patients with high BTG2 expression often have better prognoses, with BTG2 as an independent prognostic factor in ESCC [32, 33]. The results of this study are consistent with these findings: The expression of BTG2 in tumor tissues are significantly lower than in adjacent tissues; the 3-year survival rate of the high BTG2 expression group is significantly higher than that of the low expression group (72.7% vs. 48.5%), and BTG2 is an independent prognostic factor for ESCC. Additionally, the expression of BTG2 is significantly correlated with the degree of tumor differentiation, indicating that ESCC patients with low BTG2 expression tend to have poorly differentiated tumors. There are currently no reported studies linking BTG2 expression to the degree of cancer differentiation, which is consistent with the anti-proliferative effects of BTG2.

The oncogenic role of NOX2 in ESCC may be related to the regulation of BTG2 expression. Shimizu et al. found through microarray analysis that BTG2 was the fourth most upregulated gene in NOX2 knockdown ESCC cells, suggesting a negative correlation between NOX2 and BTG2 [19]. In this study, after NOX2 knockdown in ESCC cells, both mRNA and protein levels of BTG2 significantly increased, indicating that targeting NOX2 may inhibit ESCC cell proliferation by upregulating BTG2 expression. Taken together, these results demonstrate that NOX2 knockdown upregulates BTG2 expression, providing experimental evidence that NOX2 is involved in the regulation of the BTG2 gene in ESCC cells. IHC results indicate that there is no significant correlation between NOX2 and BTG2 expression. This may be due to the insufficient sample size of ESCC or the inconsistency in research methods between cellular and tissue samples. There are currently no published articles reporting on the regulatory effect of NOX2 on BTG2 and the correlation of their expression in tumor tissues. Importantly, this study, along with most literature, indicates that although the expression levels of NOX2 and BTG2 show minimal correlation with the clinical pathological factors in ESCC patients, both NOX2 and BTG2 are significantly associated with the prognosis of ESCC patients and serve as independent prognostic factors. These findings suggest that NOX2 and BTG2 may have unique value in assessing the prognosis of ESCC patients, and their combination with clinical pathological factors could provide a more accurate evaluation of prognosis for ESCC patients.

Targeting NOX2 represents a promising anticancer strategy poised for clinical translation. Emerging evidence indicates that the NOX2 inhibitor GSK2795039 attenuates doxorubicin-induced cardiotoxicity by suppressing NADPH oxidase-mediated oxidative stress and subsequent cardiomyocyte necroptosis, thereby improving myocardial remodeling and function in heart failure models [34]. With the advancing understanding of tumor biology and immune dynamics, future therapeutic paradigms will likely emphasize personalized strategies and microenvironment-focused interventions [35]. Building on this trend and our findings, the BTG2 pathway represents a promising candidate for personalized treatment design in ESCC. Restoring or enhancing BTG2 function could potentially be leveraged to tailor therapies based on the molecular profile of individual tumors, thereby improving patient outcomes. While our data demonstrate that NOX2 knockdown upregulates BTG2 expression, the precise molecular mechanism, for example, the involvement of specific transcription factors or ROS-mediated signaling pathways, requires further investigation. In addition, this study was limited to NOX2 knockdown experiments without further validation through dual inhibition of NOX2 and BTG2. Future studies will focus on identifying the intermediary players in the NOX2-BTG2 regulatory axis, and experiments employing a combinatorial approach would provide stronger evidence for the functional significance of this axis in ESCC pathogenesis.

## Conclusion

In summary, the carcinogenic role of NOX2 in ESCC may be associated with the regulation of BTG2 expression. Patients with high NOX2 expression and low BTG2 expression have poorer prognoses, and both NOX2 and BTG2 are independent prognostic factors for ESCC. These findings suggest that NOX2 and BTG2 may serve as novel biomarkers and molecular therapeutic targets for ESCC patients.

## Supplementary Information

Supplementary Material 1

## Data Availability

The clinical and pathological datasets from ESCC patients analyzed in this study are available within the manuscript and supplementary materials (Supplementary data 1).

## References

[1] Sonkin D, Thomas A, Teicher BA. Cancer treatments: past, present, and future. Cancer Genet. 2024;286–287:18–24. 38909530 10.1016/j.cancergen.2024.06.002 PMC11338712

[2] Sung H, Ferlay J, Siegel RL, et al. Global cancer statistics 2020: GLOBOCAN estimates of incidence and mortality worldwide for 36 cancers in 185 countries. CA Cancer J Clin. 2021;71:209–49. 33538338 10.3322/caac.21660

[3] He F, Wang J, Liu L, et al. Esophageal cancer: trends in incidence and mortality in China from 2005 to 2015. Cancer Med. 2021;10:1839–47. 33594825 10.1002/cam4.3647 PMC7940233

[4] Lagergren J, Smyth E, Cunningham D, et al. Oesophageal cancer. Lancet. 2017;390:2383–96. 28648400 10.1016/S0140-6736(17)31462-9

[5] Yan C, Cao W, Li J, et al. PD-1 inhibitors in advanced esophageal squamous cell carcinoma: a survival analysis of reconstructed patient-level data. Front Pharmacol. 2024;15:1408458. 39092218 10.3389/fphar.2024.1408458 PMC11291229

[6] Chen Y, Yu R, Liu Y. Combine radiotherapy and immunotherapy in esophageal squamous cell carcinoma. Crit Rev Oncol Hematol. 2023. 10.1016/j.critrevonc.2023.104115. 37633347 10.1016/j.critrevonc.2023.104115

[7] Edward Robinson A, Venkatesh Jayanthi N. Oesophageal cancer – a systemic disease, the need for targeted systemic treatments. Eur J Surg Oncol. 2024. 10.1016/j.ejso.2024.108495. 39047328 10.1016/j.ejso.2024.108495

[8] Schroder K. NADPH oxidases: current aspects and tools. Redox Biol. 2020;34:101512. 32480354 10.1016/j.redox.2020.101512 PMC7262010

[9] Grauers Wiktorin H, Aydin E, Hellstrand K, et al. NOX2-derived reactive oxygen species in cancer. Oxid Med Cell Longev. 2020;2020:7095902. 33312338 10.1155/2020/7095902 PMC7721506

[10] Vermot A, Petit-Hartlein I, Smith SME, et al. NADPH oxidases (NOX): an overview from discovery, molecular mechanisms to physiology and pathology. Antioxidants. 2021. 10.3390/antiox10060890. 34205998 10.3390/antiox10060890 PMC8228183

[11] Konate MM, Antony S, Doroshow JH. Inhibiting the activity of NADPH oxidase in cancer. Antioxid Redox Signal. 2020;33:435–54. 32008376 10.1089/ars.2020.8046 PMC7370979

[12] Trevelin SC, Shah AM, Lombardi G. Beyond bacterial killing: NADPH oxidase 2 is an immunomodulator. Immunol Lett. 2020;221:39–48. 32092360 10.1016/j.imlet.2020.02.009

[13] Wang P, Shi Q, Deng WH, et al. Relationship between expression of NADPH oxidase 2 and invasion and prognosis of human gastric cancer. World J Gastroenterol. 2015;21:6271–9. 26034362 10.3748/wjg.v21.i20.6271 PMC4445104

[14] Wang Z, Tang T, Wang S, et al. Aloin inhibits the proliferation and migration of gastric cancer cells by regulating NOX2-ROS-mediated pro-survival signal pathways. Drug Des Devel Ther. 2020;14:145–55. 32021099 10.2147/DDDT.S219247 PMC6969686

[15] Fan Z, Duan X, Cai H, et al. Curcumin inhibits the invasion of lung cancer cells by modulating the PKCalpha/Nox-2/ROS/ATF-2/MMP-9 signaling pathway. Oncol Rep. 2015;34:691–8. 26059056 10.3892/or.2015.4044

[16] Harrison IP, Vinh A, Johnson IRD, et al. Nox2 oxidase expressed in endosomes promotes cell proliferation and prostate tumour development. Oncotarget. 2018;9:35378–93. 30459931 10.18632/oncotarget.26237 PMC6226044

[17] Yang WH, Huang Z, Wu J, et al. A TAZ-ANGPTL4-NOX2 axis regulates ferroptotic cell death and chemoresistance in epithelial ovarian cancer. Mol Cancer Res. 2020;18:79–90. 31641008 10.1158/1541-7786.MCR-19-0691 PMC6942206

[18] Takiguchi K, Shimizu H, Shoda K, et al. The expression and role of NADPH oxidase 2 in colon cancer. Anticancer Res. 2023;43:2601–8. 37247898 10.21873/anticanres.16427

[19] Shimizu H, Katsurahara K, Inoue H, et al. NADPH oxidase 2 has a crucial role in cell cycle progression of esophageal squamous cell carcinoma. Ann Surg Oncol. 2022;29:8677–87. 35972670 10.1245/s10434-022-12384-5

[20] Yuniati L, Scheijen B, van der Meer LT, et al. Tumor suppressors BTG1 and BTG2: beyond growth control. J Cell Physiol. 2019;234:5379–89. 30350856 10.1002/jcp.27407 PMC6587536

[21] Liu H, P. Dilger J. Different strategies for cancer treatment: targeting cancer cells or their neighbors? Chin J Cancer Res. 2025;37:289–92. 40353083 10.21147/j.issn.1000-9604.2025.02.12 PMC12062981

[22] Yang C, Li F, Ren Y, et al. Targeting esophageal squamous cell carcinoma by combining copper ionophore disulfiram and JMJD3/UTX inhibitor GSK J4. Cancers. 2023. 10.3390/cancers15225347. 38001607 10.3390/cancers15225347 PMC10670038

[23] Jia Y, Liu Z, Dai M, et al. Decrease of 5-hydroxymethylcytosine in hepatitis B virus-related hepatocellular carcinoma: a cross-sectional study. Medicine Baltimore. 2023;102:e33943. 37266610 10.1097/MD.0000000000033943 PMC10238031

[24] Kitamoto K, Miura Y, Karnan S, et al. Inhibition of NADPH oxidase 2 induces apoptosis in osteosarcoma: the role of reactive oxygen species in cell proliferation. Oncol Lett. 2018;15:7955–62. 29731909 10.3892/ol.2018.8291 PMC5920860

[25] Lin RJ, Huang Z, Wang SL, et al. Clinicopathological and prognostic value of NADPH oxidase 2 (NOX2) in primary osteosarcoma. J Orthop Sci. 2021;26:466–72. 32402505 10.1016/j.jos.2020.04.002

[26] Ortega MA, Fraile-Martinez O, Pekarek L, et al. Oxidative stress markers are associated with a poor prognosis in patients with pancreatic cancer. Antioxidants. 2022. 10.3390/antiox11040759. 35453444 10.3390/antiox11040759 PMC9029757

[27] You X, Ma M, Hou G, et al. Gene expression and prognosis of NOX family members in gastric cancer. Onco Targets Ther. 2018;11:3065–74. 29872318 10.2147/OTT.S161287 PMC5975617

[28] Cho SY, Kim S, Son MJ, et al. Dual oxidase 1 and NADPH oxidase 2 exert favorable effects in cervical cancer patients by activating immune response. BMC Cancer. 2019;19:1078. 31706280 10.1186/s12885-019-6202-3 PMC6842485

[29] Liu Y, Han D, Ma Q, et al. Prognostic value of NOX2 as a potential biomarker for lung adenocarcinoma using TCGA and clinical validation. Mol Med Rep. 2023. 10.3892/mmr.2023.12935. 36633128 10.3892/mmr.2023.12935 PMC9879073

[30] Winkler GS. The mammalian anti-proliferative BTG/Tob protein family. J Cell Physiol. 2010;222:66–72. 19746446 10.1002/jcp.21919

[31] Kim SH, Jung IR, Hwang SS. Emerging role of anti-proliferative protein BTG1 and BTG2. BMB Rep. 2022;55:380–8. 35880434 10.5483/BMBRep.2022.55.8.092 PMC9442347

[32] Guo B, Tian Z. Mir-25 promotes metastasis of esophageal cancer by targeting BTG2. Appl Biochem Biotechnol. 2022;195:5365–78. 35239148 10.1007/s12010-022-03847-2

[33] Wang W, Guo H, Zhou S, et al. Expression and clinical significance of B cell translocation gene 2 in esophageal squamous cell carcinoma. Int J Clin Exp Pathol. 2021;14:475–83. 33936370 PMC8085815

[34] Zhang X-J, Li L, Wang A-L, et al. GSK2795039 prevents RIP1-RIP3-MLKL-mediated cardiomyocyte necroptosis in doxorubicin-induced heart failure through inhibition of NADPH oxidase-derived oxidative stress. Toxicol Appl Pharmacol. 2023. 10.1016/j.taap.2023.116412. 36764612 10.1016/j.taap.2023.116412

[35] Telang MJR, Soni B. Overview of perspectives on cancer, newer therapies, and future directions. Oncol Translational Med. 2024;10:105–9.

